# Analysis of differential membrane proteins related to matrix stiffness-mediated metformin resistance in hepatocellular carcinoma cells

**DOI:** 10.1186/s12953-023-00216-7

**Published:** 2023-09-22

**Authors:** Xiangyu Gao, Jiali Qian, Yang Zhang, Heming Wang, Jiefeng Cui, Yehong Yang

**Affiliations:** 1 Department of Endocrinology, Huashan Hospital, Fudan University, 12 Middle Urumqi Road, Shanghai, 200040 PR China; 2 Institute of Biomedical Science, Fudan University, 131 Dong’ an Road, Shanghai, 200032 PR China; 3 Department of Gastroenterology, Zhongshan Hospital, Fudan University, 136 Yi Xue Yuan Road, Shanghai, 200032 PR China; 4 Liver Cancer Institute, Zhongshan Hospital, Fudan University & Key Laboratory of Carcinogenesis and Cancer Invasion, Ministry of Education, 136 Yi Xue Yuan Road, Shanghai, 200032 PR China

**Keywords:** Hepatocellular carcinoma, Metformin, Membrane proteins, Matrix stiffness, Drug resistance, iTRAQ

## Abstract

**Background:**

Our previous work shows that increased matrix stiffness not only alters malignant characteristics of hepatocellular carcinoma (HCC) cells, but also attenuates metformin efficacy in treating HCC cells. Here, we identified differential membrane proteins related to matrix stiffness-mediated metformin resistance for better understand therapeutic resistance of metformin in HCC.

**Methods:**

Differential membrane proteins in HCC cells grown on different stiffness substrates before and after metformin intervention were screened and identified using isobaric tags for relative and absolute quantification (iTRAQ) labeling coupled with the liquid chromatography-tandem mass spectrometry (LC–MS/MS), then bioinformatic analysis were applied to determine candidate membrane protein and their possible signaling pathway.

**Results:**

A total of 5159 proteins were identified and 354 differential membrane proteins and membrane associated proteins, which might be associated with matrix stiffness-mediated metformin resistance were discovered. Then 94 candidate membrane proteins including 21 up-regulated protein molecules and 73 down-regulated protein molecules were further obtained. Some of them such as Annexin A2 (ANXA2), Filamin-A (FLNA), Moesin (MSN), Myosin-9 (MYH9), Elongation factor 2 (eEF2), and Tax1 binding Protein 3 (TAX1BP3) were selected for further validation. Their expressions were all downregulated in HCC cells grown on different stiffness substrates after metformin intervention. More importantly, the degree of decrease was obviously weakened on the higher stiffness substrate compared with that on the lower stiffness substrate, indicating that these candidate membrane proteins might contribute to matrix stiffness-mediated metformin resistance in HCC.

**Conclusions:**

There was an obvious change in membrane proteins in matrix stiffness-mediated metformin resistance in HCC cells. Six candidate membrane proteins may reflect the response of HCC cells under high stiffness stimulation to metformin intervention, which deserve to be investigated in the future.

**Supplementary Information:**

The online version contains supplementary material available at 10.1186/s12953-023-00216-7.

## Background

Increasing evidence has showed that diabetes is closely related to the occurrence and development of tumors [1–3]. The incidence of multiple tumors in patients with diabetes is higher than that in the non-diabetic population, and the risk of the incidence of liver cancer is the highest (RR ≈ 2.5) [4]. Metformin, as a first-line hypoglycemic agent for the treatment of type 2 diabetes, has recently exhibited obvious anticancer effect in a variety of solid tumors including HCC [5–8]. However, the mechanism of metformin tolerance and resistance in solid tumor remains largely unclear. Past studies on the underlying mechanism of drug resistance mainly focus on biochemical and metabolic signals within tumor microenvironment, such as DNA mutations and metabolic reprogramming, etc. [9–12]. The effect of biomechanics signals within tumor microenvironment on therapeutic resistance has been rarely mentioned. Matrix stiffening, caused by massive deposition and cross-linking of matrix proteins, is the most frequent biomechanical characteristics of solid tumor, influences and drives the development of various cancers [13–15]. HCC almost exclusively develops in the condition of chronic liver diseases such as hepatitis, liver fibrosis, cirrhosis, etc. [16, 17], and HCC patients with severe cirrhosis usually present an unfavorable prognosis [18]. In clinic, liver stiffness has gradually served as a useful indicator to predict the development and progression of HCC [19, 20]. In vitro experiments reveal that increased matrix stiffness significantly strengthens the malignant properties of HCC cells including proliferation [21], invasion and metastasis [22, 23], aerobic glycolysis [24], stemness [25], epithelial-mesenchymal transition (EMT) [26] and pre-metastatic niche formation [27]. Besides, increased matrix stiffness also enhances resistance to chemotherapeutic agents remarkably like paclitaxel, 5-FU, cisplatin and oxaliplatin, etc. [25, 28, 29]. Similarly, our latest research also supports that increased matrix stiffness attenuates metformin efficacy in treating HCC cells, disclosing a significant linkage between matrix stiffness and therapeutic resistance in HCC [30]. Cells communicate with the surrounding microenvironment via cell membrane. Membrane proteins and their associated proteins including adhesion molecules, membrane surface receptors, ion channel proteins, etc., almost contribute to all kinds of physiological and pathological activities of cells. Composition and expression of membrane proteins usually determines the morphology of cells and their migration abilities. Membrane protrusion formation and cell morphological changes are the initial steps of invasion and migration of cancer cells, and the membrane proteins in HCC cells closely correlate with their malignant characteristics of liver cancer such as proliferation, invasion, and metastasis [31–33]. Increased matrix stiffness can also obviously alter the morphology of tumor cells [34, 35], improve their migration abilities [26], affect membrane proteins’ expression and distribution [36, 37]. However, little is known about the changes and functions of membrane proteins in matrix stiffness-mediated metformin resistance in HCC cells.

Here, we used isobaric tags for relative and absolute quantification (iTRAQ) labeling coupled with LC–MS/MS to identify differential membrane proteins related to matrix stiffness-mediated metformin resistance in HCC cells for better understand the mechanism of matrix stiffness-caused metformin resistance.

## Methods

### Preparation of gel substrates with stiffness 6,10,16 kPa in vitro

An in vitro gel-based culture system with tunable stiffness was established as the method described previously except for the coated matrix protein [21]. The established gel substrates with a diameter of 6 cm (stiffness 6,10 and16 kPa) were coated with 320 µl fibronectin (FN) solution (0.17 mg/ml). For further details, please refer to supplementary files attached (Additional file 1: Table S1).

### Cell culture and metformin intervention

MHCC97H cells, a type of human HCC cell lines with high metastatic potential, was established at the Liver Cancer Institute of Fudan University. MHCC97H cells were cultured in Dulbecco’s Modified Eagle’s Medium (DMEM, Gibco, USA) supplemented with 10% fetal bovine serum (FBS, Biowest, South America Origin) and 1% penicillin/streptomycin (Gibco, USA). Approximately 1 × 10^6^ cells in 0.3 ml of culture medium were placed evenly onto the surface of FN-coated gel substrates (diameter in 6 cm) and cultured for 2 h at room temperature. Subsequently, 10 ml culture medium was carefully added into culture dish. The cells were transferred to the incubator(37 °C, 5% CO2)for 48 h culture. The cells grown on different stiffness substrates were collected by a cell scraper.

1 mol/L of metformin (Sigma-Aldrich, St. Louis, MO, USA) was prepared as a stock solution for storage. Intervention concentration of metformin solution was determined based on a dose–response curve of metformin in HCC cells [30], that is 27 mM in MHCC97H cells.

### Membrane protein extraction

Membrane proteins were extracted from HCC cells using Membrane Protein Extraction Kit (Thermo Fisher, USA) according to the manufacturer’s protocol. In briefly, the cells were collected and permeated with Permeabilization Buffer, a mild detergent, to release soluble cytoplasmic proteins. A second detergent Solubilization Buffer was used to solubilize and extract membrane and membrane-associated proteins. Protease and phosphatase inhibitors (Thermo Fisher, USA) were added into the Permeabilization and Solubilization Buffers to avoid protein degradation. The concentration of the extracted membrane proteins was measured using BCA Protein Assay Kit (Beyotime, China).

### iTRAQ labeling coupled with LC–MS/MS detection

The quality and concentration of the extracted membrane proteins were confirmed to meet the requirement of subsequent experiment. Briefly, 100 µg membrane protein samples of each group were taken into new Eppendorf (EP) tubes, which is relatively small centrifuge tubes, and the volume was supplemented to a total volume of 150 µl with lysate, respectively. Tris(2-carboxyethyl)phosphine (TCEP) (Thermo Fisher, USA) was added at final concentration of 10 mM and reacted for 60 min at 37 °C. Then we added 40 mM iodoacetamide (Sigma, USA) to each of the tubes and allowed to react for 40 min at room temperature, which also need to be protected from light. Pre-cooled acetone (CHINA SINOPHARM INTERNATIONAL (SHANGHAI) CO., LTD., China) was added to each tube at a ratio of 6:1 acetone: sample volume, and precipitated at -20 °C for 4 h. Centrifuged at 10,000 × g for 20 min and discarded the supernatant to remove the pellet. The precipitated sample was fully dissolved with 100 µl 100 mM tetraethyl ammonium bromide (TEAB) (Santa Cruz, USA). In the process above, the extracted protein completed reduction and alkylation. Then Trypsin (Hualishi Technology, China) was added to with ratio of enzyme to protein, 1:50 to enzymolyze the equal amounts of protein overnight at 37 °C. Following Trypsin digestion, peptides were evacuated by vacuum pump and then reconstituted with TEAB (0.4 M). The iTRAQ reagent (AB Sciex, USA) stored at -20 °C was brought to room temperature, centrifuged and isopropanol was added, followed by vortex centrifugation, then 1 tube of iTRAQ reagent was added to every 100 µg of peptide and incubated at room temperature for 2 h. And peptides were labeled with iTRAQ reagent as follows: NC-L, iTRAQ 113; NC-M, iTRAQ 114; NC-H, iTRAQ 115; Met-L, iTRAQ 116; Met-M, iTRAQ 117; Met-H, iTRAQ 118. Then, added 50 µl ultrapure water to each of the above mixture solution and placed at room temperature for 30 min. Next, The labeled peptides were mixed equally into a new tube, and dried by vacuum concentrator. Peptide samples reconstituted with Ultra-High Performance Liquid Chromatography (UPLC) loading buffer were subjected to high pH liquid phase separation using a reversed-phase C18 column (Waters, USA). UPLC loading buffer is consisted of A and B. Buffer A is 2% acetonitrile (adjusted to pH 10 with liquid ammonia) and buffer B is 80% acetonitrile (adjusted to pH 10 with liquid ammonia). UV detection wavelength was 214 nm. Flow rate was 200 µl/min. Gradient was 50 min. A total of 36 fractions were collected according to peak pattern and time, combined into 12 fractions, concentrated by vacuum centrifugation, dissolved with mass spectrometry loading buffer, and then analyzed on LC-MS/MS system (Thermo, USA). Full scan resolution was 70,000. MS/MS resolution was 17,500. Parent ion scanning range was 300–1800 m/z. The standardized collision energy value was 30 eV. The total running time was 120 min.

### Database searching and bioinformatics

Data were acquired automatically on LC-MS/MS system by data dependent acquisition (DDA) mode. Swiss Prot-Human database was selected because HCC cells were human-derived. The raw file was submitted to the proteome discoverer software (version 1.4) during the database searching, selected the established database, and then performed the database search. False Discovery Rate (FDR) ≤ 0.01. Ratios of the 116/113, 117/114 and 118/115 tags from the iTRAQ-labeled peptides were calculated using MATLAB (version 2019b, MathWorks, USA). Fold changes more than 1.5 or less than 0.67 were set as cutoff values to screen significant differences in protein expression. *P* < 0.05 in three replicates was considered to be statistically significant for protein quantification.

The screened data were analyzed using bioinformatics. We further predicted the protein-protein interactions (PPI) network and signaling pathways which the candidate differentially expressed membrane proteins might be mediated or included using Ingenuity Pathway Analysis (IPA) software. R-Package clusterProfiler was used to analyze the target genes for K-means and Gene Ontology (GO) enrichment analysis, which was also used to map the volcano plots.

### Coomassie brilliant blue staining and western blot

For Coomassie brilliant blue staining, approximately 20 µg proteins were loaded and separated by sodium dodecyl sulphate-polyacrylamide gel electrophoresis (SDS-PAGE), then placed the gel in an appropriate amount of Coomassie blue stain to ensure that the stain adequately covered the gel. Next, stained at room temperature for 1 h with gentle shaking on a shaker. Discarded the dye solution, and added an appropriate amount of Coomassie brilliant blue destaining solution, ensured that the destaining solution covered the gel, shaked slowly on a shaker at room temperature until the blue background was completely removed and the stained protein bands were clear. Finally, stained protein bands were photographed. Similarly, when performing western blot, approximately 20 µg proteins were loaded and separated by SDS-PAGE, and then transferred onto a polyvinylidene difluoride (PVDF) membrane (Millipore, USA). Subsequently, the PVDF membrane was blocked in 1×Tris buffered saline (TBS)-Tween ( Sangon Biotech, China) containing 5% fat-free milk, then incubated overnight at 4 °C with the following primary antibodies against human β-Tubulin (1:5000, Affinity), Na-K ATPase (1:1000, Abcam), Filamin-A (1:10000, Abcam), Myosin IIA (1:1000, Abcam), EEF2 (1:10000, Abcam), Moesin (1:20000, Abcam), ANXA2 (1:1000, Abcam), TIP-1 (1:1000, Abcam). Afterwards, the membrane was incubated with Horseradish Peroxidase (HRP)-conjugated secondary antibody (1:5000, Proteintech) on a shaker at room temperature for 1 h. Finally, the target protein bands were visualized by an electrochemiluminescence kit (Tanon, China) and detected using Molecular Imager (Bio-Rad, Hercules, CA).

### Statistical analysis

Data analysis was performed using SPSS 20.0 statistical software (SPSS Inc., Chicago, IL, USA). Quantitative variables were expressed as mean ± standard deviation (SD) and statistical analysis was using Student’s t-test. A *P* < 0.05 (two-tailed) was considered statistical significance.

## Results

### Identification and categorization of differential membrane proteins

**Fig. 1:**
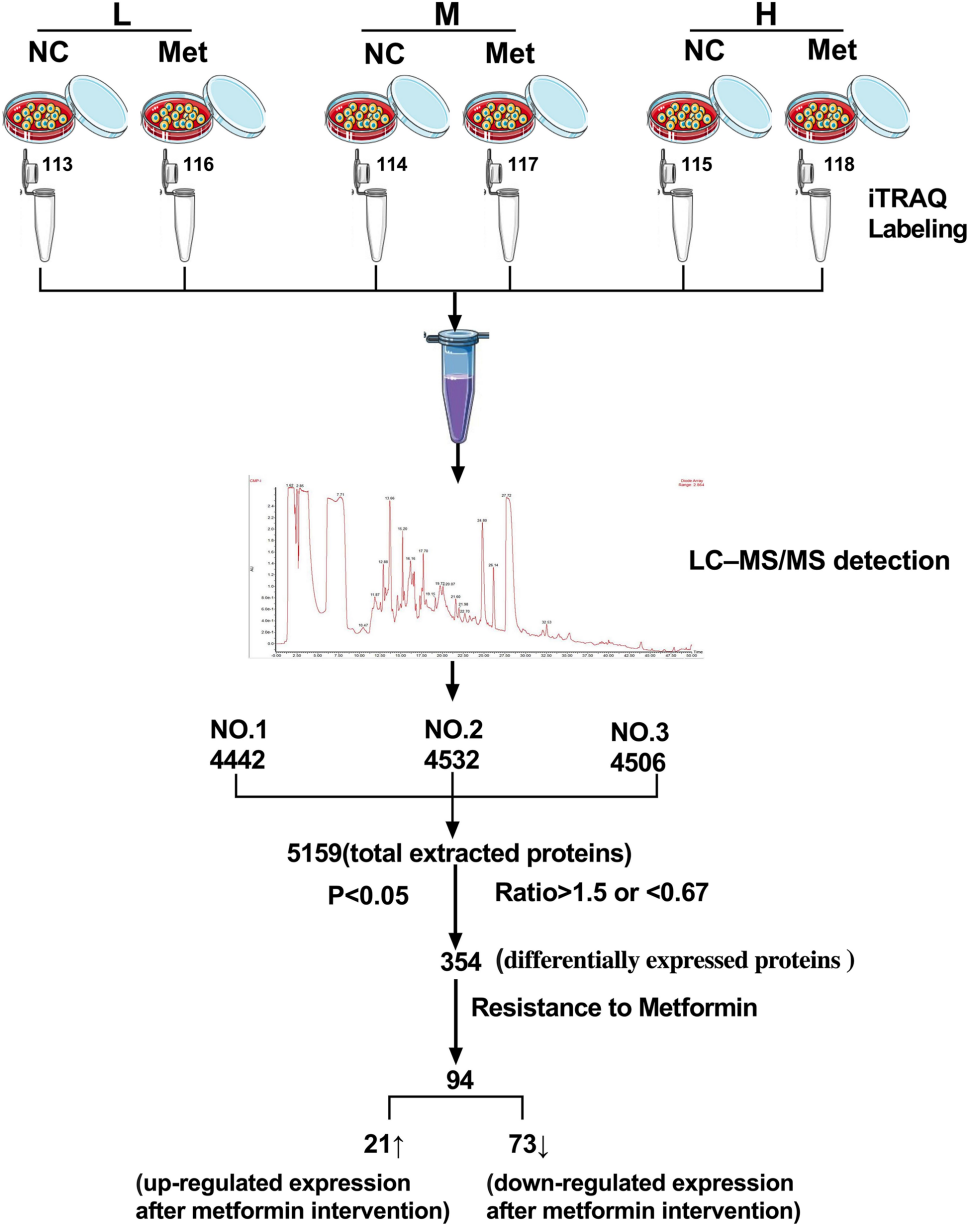
The workflow chart based on cell culture substrates with tunable stiffness in vitro. L: low stiffness; M: medium stiffness; H: high stiffness. NC refers to non-metformin intervention; Met refers to metformin intervention. iTRAQ tags 113, 114, 115, 116, 117 and 118 represent NC-L, NC-M, NC-H, Met-L, Met-M and Met-H, respectively

Increased matrix stiffness not only alters obviously morphology and spreading area of HCC cells [21, 35], but also attenuates antitumor effects of therapeutic drugs including metformin [28–30]. These findings led us to speculate that the expression and distribution of membrane proteins might participate in matrix stiffness-mediated metformin resistance. Using same intervention system as the method reported previously [30], we developed in vitro matrix stiffness-mediated metformin resistance cell models for differential membrane proteomics analysis. A detailed flow chart of differential membrane proteomics analysis was shown in Fig. 1. According to the presence or absence of metformin, we divided HCC cells grown on low (6 kPa), medium (10 kPa) and high (16 kPa) stiffness substrates into NC group and Met group. Firstly, we evaluate the quality of the extracted membrane proteins and cytoplasmic protein by polyacrylamide gel electrophoresis staining analysis and internal reference protein analysis. Molecular weight distribution of the extracted membrane proteins in Coomassie brilliant blue staining from HCC cells was consistent with the pattern of membrane protein. Additionally, the extracted membrane proteins from HCC cells had the expression of Na-K ATPase, but almost no expressions in β-tubulin. On the other hand, the extracted cytoplasmic proteins exhibited high expression of β-tubulin, but no expression in Na-K ATPase. These above results suggested that the extracted membrane proteins and membrane junction proteins were qualified for subsequent differential membrane protein analysis (Additional file 2: Figure S1).

**Fig. 2:**
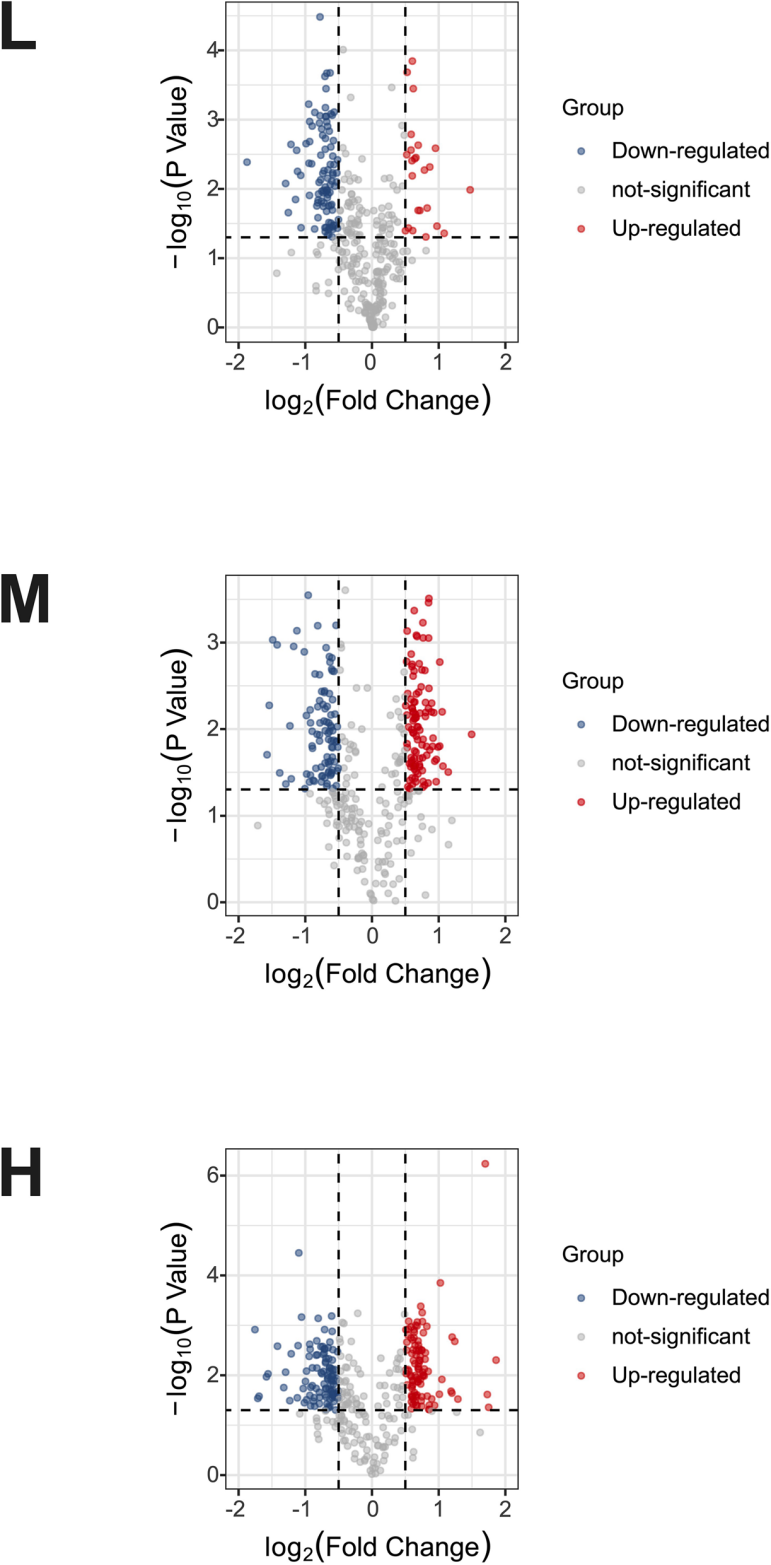
The volcano plot of differentially expressed membrane proteins. Each point in the volcano map represents one protein molecule. Red dots indicate the upregulated proteins after metformin intervention, blue dots represent the downregulated proteins, and the black dots represent nondifferential expressed proteins. The abscissa represents the logarithmic value of the fold expression of a certain protein in the two samples before and after metformin intervention

**Fig. 3:**
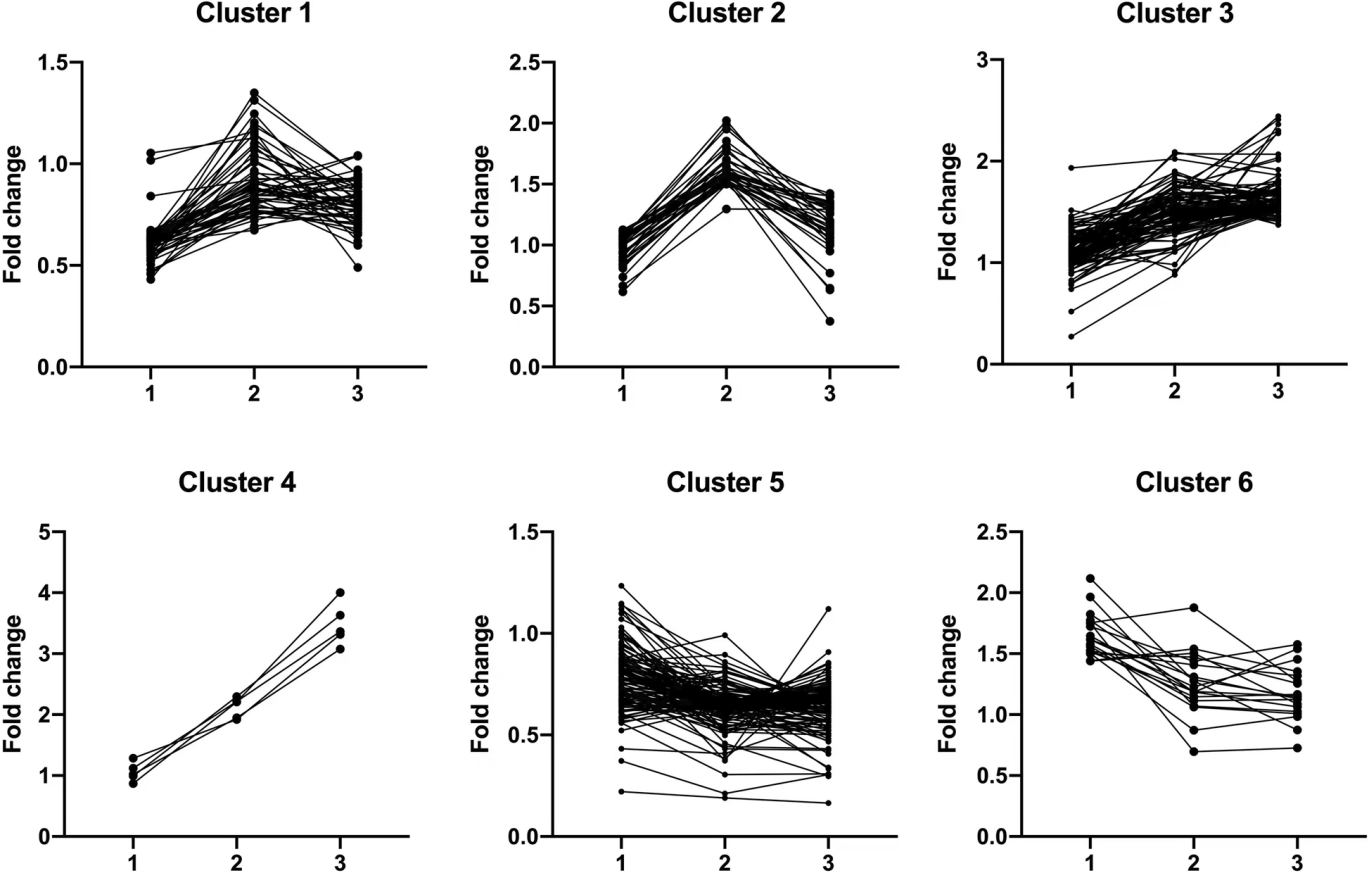
K-means clustering analysis of 6 expression patterns with different trend plots according to the relative expression levels of proteins at different groups. "1" refers to Met-L/NC-L; "2" refers to Met-M/NC-M; and "3" refers to Met-H/NC-H. Maximum Normalization was performed

Subsequently, we used a quantitative proteomics technology iTRAQ labeling coupled with LC-MS/MS to screen differential membrane proteins related to matrix stiffness-mediated metformin resistance in HCC cells. A total of 5159 membrane proteins and membrane associated proteins were identified (Additional file 3: Table S2), and among them, 354 differential proteins, which might be associated with matrix stiffness-mediated metformin resistance, were discovered by defining fold-change > 1.5 or < 0.67 and *P* < 0.05 as a threshold (Additional file 4: Table S3). The volcano plot of differential membrane proteins and membrane associated proteins from HCC cells grown on 6 kPa (L), 10 kPa (M), and 16 kPa (H) substrates after metformin intervention (Fig. 2) showed that 20 upregulated proteins and 86 downregulated proteins in group L, 89 upregulated proteins and 84 downregulated proteins in group M, and 91 upregulated proteins and 93 downregulated proteins in group H. Based on the expression pattern of membrane proteins grown on different stiffness substrates before and after metformin intervention, we further clustered these differential membrane proteins and membrane associated proteins into 6 types of expression patterns using K-means cluster analysis (Fig. 3), and found that twelve typical expression patterns (Types I to VI of up-regulation group;Types A to F of down-regulation group) could reflect the response of HCC cells grown on different stiffness substrates to metformin intervention. We present the raw data for this section in Additional file 5: Figure S2 and Additional file 6: Figure S3. Importantly, the expression level changes of 94 differential membrane proteins (Types II, V, VI in Figure S2 and Types A, C, D in Figure S3) in HCC cells grown on the higher-stiffness substrate after metformin intervention were obviously weaker than those on the lower-stiffness substrate (Table 1), indicating that there exists a close linkage between these candidate membrane proteins and matrix stiffness-mediated metformin resistance.

**Table 1:** Typical membrane proteins screened based on resistance to the membrane inhibitory effect induced by high matrix stiffness

ProteinName	GeneName	116/113	117/114	118/115
Prelamin-A/C	LMNA	1.44066	1.503553	1.085137
Small nuclear ribonucleoprotein E	SNRPE	1.442228	1.540465	1.354483
Insulin-like growth factor-binding protein 1	IGFBP1	1.934701	2.022608	1.862529
Cytochrome c	CYCS	1.055029	2.020912	1.032612
Rho GTPase-activating protein 17	ARHGAP17	1.460284	1.885713	1.442544
Calcium/calmodulin-dependent protein kinase type II subunit delta	CAMK2D	1.380145	1.73068	1.372204
Bcl-2-binding component 3	BBC3	1.753446	1.876528	1.271211
Lamina-associated polypeptide 2, isoform alpha	TMPO	1.521199	1.408843	1.319708
Keratin, type I cytoskeletal 17	KRT17	1.823483	1.285828	1.145455
Importin subunit alpha-5	KPNA1	1.500398	1.471094	1.256237
Nuclear mitotic apparatus protein 1	NUMA1	1.964777	1.070844	1.026603
Programmed cell death protein 4	PDCD4	1.558858	1.062319	1.008753
Zinc finger HIT domain-containing protein 2	ZNHIT2	2.118072	1.276212	0.873924
Lysine-specific demethylase 2A	KDM2A	1.645833	1.185506	1.064857
Chromodomain-helicase-DNA-binding protein 4	CHD4	1.616529	1.310324	1.129761
Ubiquitin-like domain-containing CTD phosphatase 1	UBLCP1	1.514853	1.189058	1.150809
Importin subunit alpha-3	KPNA4	1.578245	1.115025	1.123152
Complement decay-accelerating factor	CD55	1.724473	1.438625	1.576691
Sodium/myo-inositol cotransporter	SLC5A3	1.615669	1.228817	1.454788
TFIIH basal transcription factor complex helicase XPB subunit	ERCC3	1.526915	1.146046	1.165517
High affinity cationic amino acid transporter 1	SLC7A1	1.774459	1.184333	1.540541
Annexin A2	ANXA2	0.61652	0.899812	0.86238
Pyruvate kinase PKM	PKM	0.58267	0.953339	0.9183
Moesin	MSN	0.592999	0.965812	0.718781
Peptidyl-prolyl cis–trans isomerase A	PPIA	0.62565	0.790193	0.735385
Na( +)/H( +) exchange regulatory cofactor NHE-RF1	SLC9A3R1	0.620426	0.888193	0.711522
40S ribosomal protein SA	RPSA	0.587755	0.767195	0.699355
Ubiquitin-40S ribosomal protein S27a	RPS27A	0.651907	0.823816	0.771223
Destrin	DSTN	0.534129	0.845319	0.834041
Cofilin-1	CFL1	0.430929	1.067309	0.864193
LIM and SH3 domain protein 1	LASP1	0.630703	0.832025	0.796359
Fascin	FSCN1	0.665624	0.875154	0.806212
General vesicular transport factor p115	USO1	0.633543	1.006682	0.777284
Cysteine and glycine-rich protein 1	CSRP1	0.630921	1.09839	0.787614
Calponin-2	CNN2	0.651804	0.87804	0.813255
Plasminogen activator inhibitor 1 RNA-binding protein	SERBP1	0.622013	0.906532	0.760247
Protein S100-A6	S100A6	0.607469	1.085248	0.689778
40S ribosomal protein S20	RPS20	0.648542	1.040342	0.885948
Protein S100-A10	S100A10	0.53718	1.349493	0.94674
Fatty acid-binding protein, epidermal	FABP5	0.552082	0.764246	0.680111
Cdc42 effector protein 4	CDC42EP4	0.569404	0.9141	0.787523
Methionine aminopeptidase 2	METAP2	0.523168	1.185742	0.885365
Clustered mitochondria protein homolog	CLUH	0.638349	0.760548	0.749095
Programmed cell death protein 5	PDCD5	0.561837	0.804717	0.652728
Tax1-binding protein 3	TAX1BP3	0.637478	1.147379	0.826159
28S ribosomal protein S21, mitochondrial	MRPS21	0.586635	0.711005	0.609355
Apoptosis-associated speck-like protein containing a CARD	PYCARD	0.604483	0.765976	0.701165
Early endosome antigen 1	EEA1	0.572098	0.869641	0.785589
Tetratricopeptide repeat protein 17	TTC17	0.629422	0.783653	0.776334
Exocyst complex component 5	EXOC5	0.628536	0.772019	0.733332
Thioredoxin domain-containing protein 17	TXNDC17	0.550895	0.739947	0.711344
Malignant T-cell-amplified sequence 1	MCTS1	0.593491	1.018201	0.726474
Keratin, type II cuticular Hb6	KRT86	0.569451	0.607246	0.570316
Myotrophin	MTPN	0.642847	1.312341	0.948826
Thymosin beta-10	TMSB10	0.570746	1.245873	0.678481
Interleukin-1 alpha	IL1A	0.615046	0.695885	0.661868
Sulfiredoxin-1	SRXN1	0.58674	0.637876	0.590507
SH3 domain-binding glutamic acid-rich-like protein 3	SH3BGRL3	0.476877	0.627616	0.619659
Filamin-A	FLNA	0.624602	0.843308	0.97049
Glucose-6-phosphate 1-dehydrogenase	G6PD	0.616705	0.800959	0.938911
Myosin-9	MYH9	0.523293	0.747035	0.853047
Fatty acid synthase	FASN	0.582805	0.659283	0.722716
Elongation factor 2	EEF2	0.599412	0.870375	0.918953
Talin-1	TLN1	0.600162	0.809829	0.902139
40S ribosomal protein S21	RPS21	0.603461	0.922122	0.928002
Transgelin-2	TAGLN2	0.461376	0.84302	0.886943
Cystatin-B	CSTB	0.478538	0.692575	0.769809
Flavin reductase (NADPH)	BLVRB	0.65842	0.72311	0.734823
Nucleosome assembly protein 1-like 1	NAP1L1	0.457118	0.811807	0.891115
Golgin subfamily A member 1	GOLGA1	0.620774	0.686481	0.714003
Programmed cell death protein 6	PDCD6	0.646062	0.745869	0.84697
Hepatocyte growth factor-regulated tyrosine kinase substrate	HGS	0.633505	0.901127	1.041291
Ubiquitin-conjugating enzyme E2 variant 1	UBE2V1	0.615346	0.864989	0.933444
Exocyst complex component 6	EXOC6	0.652659	0.671117	0.719448
Macrophage migration inhibitory factor	MIF	0.6369	0.926661	0.927719
Exocyst complex component 2	EXOC2	0.522173	0.639032	0.69052
cAMP-dependent protein kinase type I-alpha regulatory subunit	PRKAR1A	0.62013	0.626398	0.744376
Exocyst complex component 1	EXOC1	0.577106	0.684352	0.72695
Nucleosome assembly protein 1-like 4	NAP1L4	0.613517	0.673389	0.839352
Alanine–tRNA ligase, cytoplasmic	AARS	0.563899	0.720974	0.853792
E3 ubiquitin-protein ligase MARCH6	6-Mar	0.637299	0.650759	0.83165
Histone H3.1	HIST1H3A	0.663002	0.851717	1.037266
Sequestosome-1	SQSTM1	0.573854	0.57978	0.588272
Peptidyl-prolyl cis–trans isomerase FKBP1A	FKBP1A	0.419217	0.426265	0.572027
Tubulin alpha chain-like 3	TUBAL3	0.75047	0.593122	0.753641
28S ribosomal protein S11, mitochondrial	MRPS11	0.647251	0.637203	0.669923
39S ribosomal protein L14, mitochondrial	MRPL14	0.724559	0.615447	0.740257
Protein phosphatase PTC7 homolog	PPTC7	0.760775	0.642389	0.774724
Toll-interacting protein	TOLLIP	0.66985	0.656658	0.762507
Thrombospondin-1	THBS1	0.432559	0.40842	0.580485
Spondin-1	SPON1	0.607419	0.383333	0.834185
Midkine	MDK	0.624462	0.609669	0.855459
Tumor necrosis factor receptor superfamily member 1A	TNFRSF1A	0.691871	0.624775	0.729306
Serpin B7	SERPINB7	0.407513	0.343795	0.460286

iTRAQ tags 113, 114, 115, 116, 117 and 118 represent NC-L, NC-M, NC-H, Met-L, Met-M and Met-H, respectively

### Validation of differential expression patterns of candidate membrane proteins

**Fig. 4:**
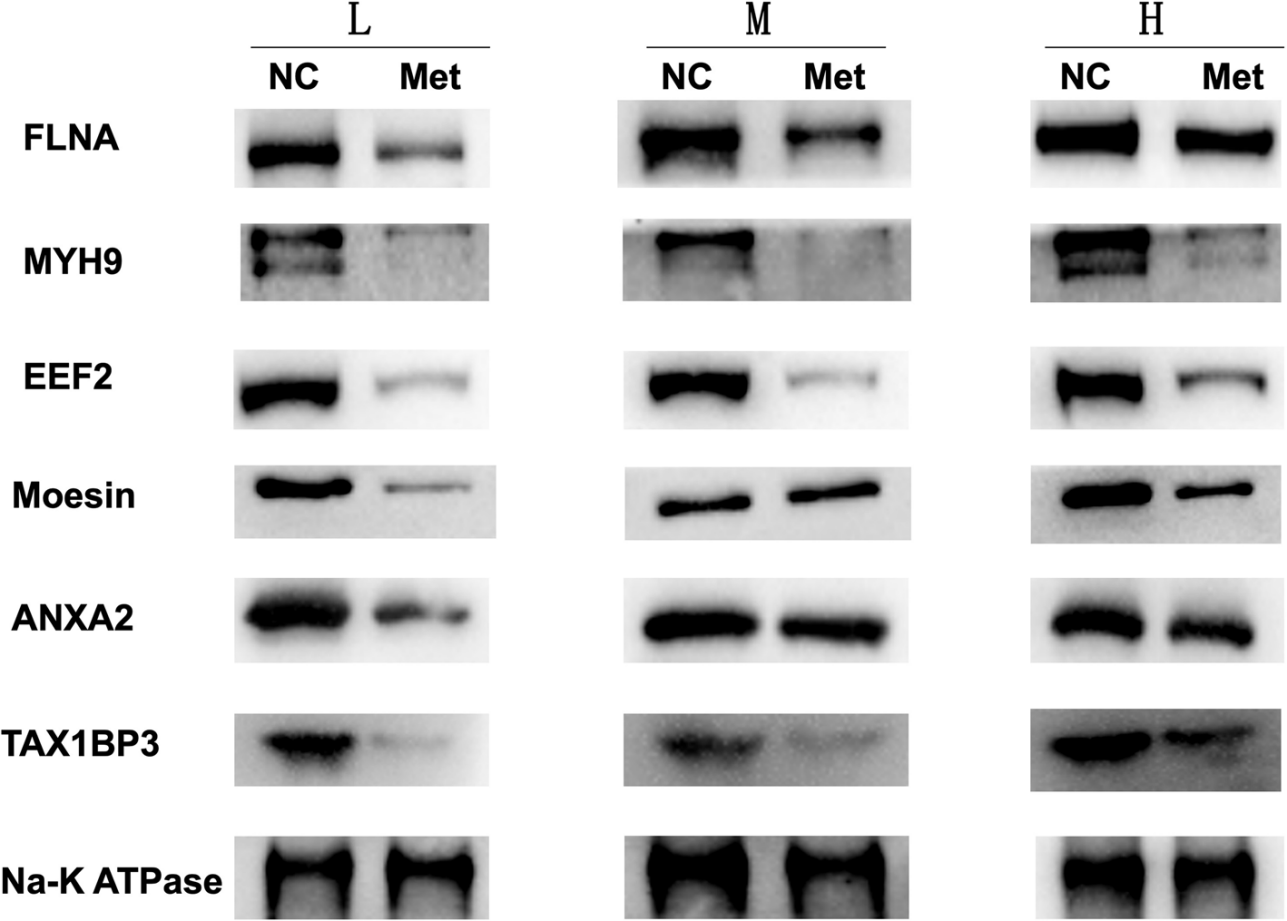
Validation of differential expression patterns of selected membrane proteins. The expressions of FLNA, MYH9, EEF2, Moesin, ANXA2 and TAX1BP3 were all downregulated in HCC cells grown on different stiffness substrates after metformin intervention, While the degree of decrease was weakened as matrix stiffness was increased, consistent with the iTRAQ results

Due to their impacts on the malignant characteristics of HCC cells, some of candidate membrane proteins mentioned above like Annexin A2 (ANXA2), Filamin-A (FLNA), Moesin (MSN), Myosin-9 (MYH9), Elongation factor 2 (eEF2) and Tax1-binding Protein 3 (TAX1BP3) were further selected as the potential target proteins for future function analysis. As shown in Fig. 4, the expressions of the above membrane proteins were all downregulated in HCC cells grown on different stiffness substrates after metformin intervention. On the other hand, the degree of decrease was obviously weakened as matrix stiffness was increased. These results were in agreement with the results of iTRAQ analysis, indicating that these candidate membrane proteins might contribute to matrix stiffness-mediated metformin resistance in HCC.

### Prediction of signaling pathways in which the validated proteins above may be participated

**Fig. 5:**
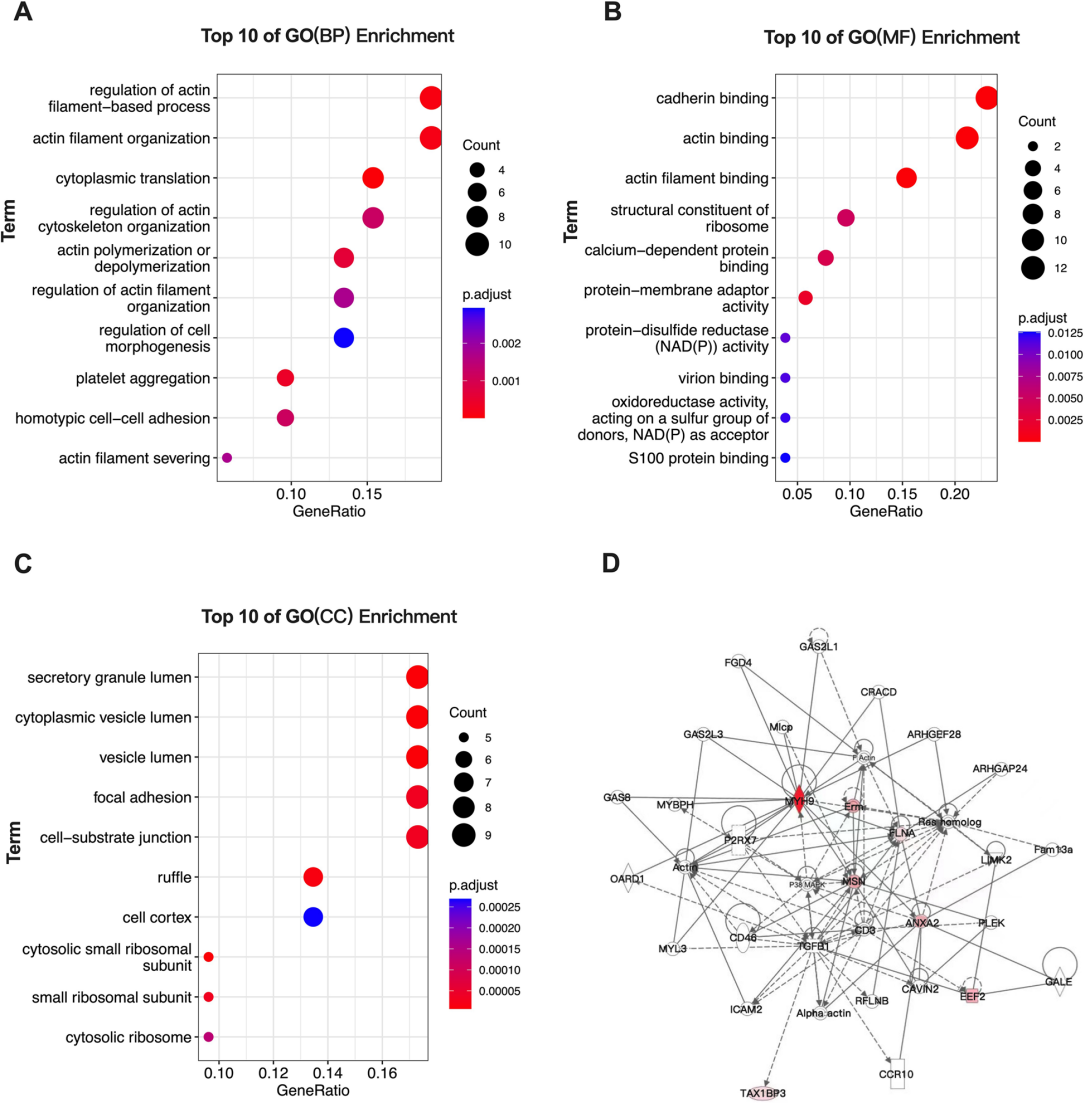
Gene Ontology (GO) enrichment analysis of genes in cluster 1 and protein–protein interaction (PPI) network construction of the six candidate membrane proteins. **A** The top 10 GO (biological process, BP) enrichment terms of the cluster 1 proteins; **B** The top 10 GO (molecular function, MF) enrichment terms of the cluster 1 proteins; **C** The top 10 GO (cellular component, CC) enrichment terms of the cluster 1 proteins; **D** PPI network of the six candidate membrane proteins involved in predicted pathways

The expression pattern of membrane proteins like ANXA2, FLNA, MSN, MYH9, eEF2 and TAX1BP3 indicated that they might be all involved in matrix stiffness-mediated metformin resistance in HCC. We further predicted the protein-protein interactions (PPI) network and signaling pathways which the above membrane proteins might be mediated or included using Ingenuity Pathway Analysis (IPA) software. R-Package clusterProfiler was used to analyze the target genes for Gene ontology (GO) enrichment analysis [38]. Coincidentally, the candidate membrane proteins mentioned above were all belonged to the cluster 1 in Fig. 3. Here, we analyzed them for GO enrichment analysis, and some potential pathways were also shown in Fig. 5. As showed in Fig. 5A, we found that cytoplasmic translation, regulation of actin filament-based process, actin filament organization, platelet aggregation, actin polymerization or depolymerization, homotypic cell-cell adhesion, regulation of actin cytoskeleton organization, actin filament severing, regulation of actin filament organization and regulation of cell morphogenesis were enriched in differential expressed proteins of the BP terms. Of the MF terms, cadherin binding, actin binding, actin filament binding, protein-membrane adaptor activity, calcium-dependent protein binding, structural constituent of ribosome, protein-disulfide reductase (NAD(P)) activity, virion binding, oxidoreductase activity, acting on a sulfur group of donors, NAD(P) as acceptor and S100 protein binding were enriched (Fig. 5B). Of the CC terms, cytosolic small ribosomal subunit, secretory granule lumen, cytoplasmic vesicle lumen, vesicle lumen, ruffle, small ribosomal subunit, focal adhesion, cell-substrate junction, cytosolic ribosome and cell cortex were enriched (Fig. 5C). Besides, PPI network of the six candidate membrane proteins involved in important pathways were illustrated in Fig. 5D. Whether these predicted signal pathways are involved in matrix stiffness-mediated metformin resistance in HCC needs to be confirmed in our future work.

## Discussion

Membrane proteins and their associated proteins almost participate in all physiological and pathological activities of the cells such as cell adhesion, receptor signal transduction, metabolic exchange, ion transport, protein/drug macromolecular transport, immune response, etc. Additionally, they also serve as biomarkers for early diagnosis or prognosis of various cancers, or as the drug targets for evaluating pharmaceutical responses in human malignancies. Increased matrix stiffness can obviously alter the morphology of tumor cells [34, 35] and strengthen their malignant properties [26, 39], indicating that there exists a significant correlation among matrix stiffness, membrane associated protein and malignant behaviors. However, little is known about whether changes and remodeling of matrix stiffness-induced membrane proteins modulates the malignant characteristics of cancer cells. HCC ranks sixth in incidence and fourth in mortality among malignant tumors [40]. The main treatment for HCC is surgery, supplemented by radiotherapy, chemotherapy and transcatheter arterial chemoembolization (TACE). Typical chemotherapeutic drugs such as cisplatin, oxaliplatin, 5-fluorouracil (5-FU), and PD-1/PDL1 monoclonal antibody play an important role in inhibiting cancer progression and prolonging survival. As traditional hypoglycemic agent for type 2 diabetes, metformin alone or combined other drugs, recently exhibits obvious antitumor effect in HCC [41–43]. Biochemical or metabolic factors-mediated drug resistance have been well documented in tumor [44, 45]. However, biomechanical signal-caused therapeutic resistance remains largely unexplored. Recently, our work suggests that higher matrix stiffness significantly attenuates the inhibitory effect of metformin on HCC invasion and metastasis, and PTEN/PI3K/Akt/MMPs pathway contributes to matrix stiffness-mediated metformin resistance [30], highlighting a significant role of biomechanical signal in metformin intervention resistance in HCC. Membrane proteins deliver outside stimulating signals into cells, subsequently influence their biological function. Integrin β1 acts as a “bridge molecule” to transduce extracellular mechanical signal into the cell [21, 46]. Membrane proteins are also involved in stiffness-regulated biological behaviors such as tumor cell proliferation and differentiation, migration and invasion [37, 47, 48]. On the other hand, changes in the cell membrane composition of HCC cells could induce chemoresistance and affect tumor chemosensitivity. Transmembrane glycoprotein FAS ligand (FASLG) is negatively targeted by micoRNA-21-5p, and high expression of micoRNA-21-5p induces cisplatin resistance in HCC cells by inhibiting FASLG expression levels [49]. ABC transporters, also known as ATP-binding cassette proteins, is one of the conventional mechanisms of multidrug resistance (MDR), and effective MDR modulators are regarded as the key for enhancing tumor chemosensitivity. Quercetin inhibits ABCB1, ABCC1, and ABCC2 protein expression through the Wnt/β-catenin pathway to improve the chemosensitivity of HCC cells [50].

In addition, metformin, through the AMPK-CEBPβ pathway, targets and inhibits the expression of the HCC cell surface membrane protein CD133, suggesting that metformin regulates the expression of membrane proteins and plays a cancer-suppressing role [51]. Metformin combined with epigallocatechin gallate (EGCG) significantly reduced the expression level of the membrane interstitial protein phosphatidylinositol proteoglycan-3 (GPC3) in HCC cells and inhibited their proliferation [52]. These studies suggest that membrane protein molecules of HCC cells might be the targets of chemotherapeutic drugs including metformin, further play a role in tumor suppression.

In order to explore the changes of membrane proteins in matrix stiffness-mediated metformin resistance in HCC cells, here, we comparatively analyzed the changes of membrane proteins in HCC cells grown on variable stiffness substrates before and after metformin intervention. By defining fold-change > 1.5 or < 0.67 and *P* < 0.05 as a threshold, we first screened 354 differential expressed membrane proteins and membrane associated proteins among the total 5159 proteins in HCC cells before and after metformin intervention grown on different stiffness substrates. According to their expression patterns of group L, M and H, we further obtained 94 candidate membrane proteins related to matrix stiffness-mediated metformin resistance. Bioinformatic analysis showed that the expressions of 94 candidate membrane proteins were attenuated in HCC cells grown on high stiffness substrate compared with the control cells on low stiffness substrate. Among the 94 proteins mentioned above, we need to select out representative molecules for further validation. Representative proteins tended to been previously reported to regulate the malignant biological behaviors or chemosensitivity of cancer. Thus, six of these membrane proteins including Annexin A2 (ANXA2), Filamin-A (FLNA), Moesin (MSN), Myosin-9 (MYH9), Eukaryotic binding Elongation factor 2 (eEF2), Tax1-Protein 3 (TAX1BP3; TIP-1) were selected to validate their expression pattern. Since the expression patterns of the above six membrane protein molecules were consistent with the proteomics analysis, these candidate membrane proteins might contribute to matrix stiffness-mediated metformin resistance in HCC.

These six membrane proteins have been previously documented to be associated with malignant tumor progression and chemosensitivity. ANXA2, which is highly expressed in HCC tissues, interacts with ELMO1 to promote HCC cell chemotaxis and metastasis [53]. ANXA can be used as a supplementary serological marker for early diagnosis of HCC, and ANXA combining with AFP achieve a sensitivity of 87.4% for screening of early HCC [54]. Besides, ANXA2 contributes to therapeutic resistance in several tumors [55, 56]. Analysis of 113 patients with HCC after resection indicate a correlation between FLNA and HCC recurrence rate. FLNA expression could predict the early recurrence of HCC after hepatectomy and contribute to postoperative follow-up [57]. Additionally, a comparative proteomics analysis suggests that FLNA can be used as a potential marker for HCC progression [58]. Moreover, FLNA also serves as a predictor of chemoresistance in some cancers such as colorectal cancer, cervical cancer, etc. [59, 60]. Moesin, a member of the ezrin-radixin-moesin (ERM) family, is involved in the regulation of cell adhesion, polarity, and migration through cross-linking between plasma membrane proteins and the actin cytoskeleton, and has been found to play a key role in hepatic stellate cell activation and liver fibrogenesis [61]. Overexpression of miR-200c negatively regulates Moesin expression to inhibit the proliferation and invasion of glioma cells, Moesin is highly expressed in glioma specimens, and Moesin promotes glioma cell development [62]. Moesin participates in regulation of breast cancer therapeutic resistance [63, 64]. A proteomic study on colorectal liver metastases demonstrates that MYH9 overexpression was associated with shorter overall survival and disease-free survival, indicating that MYH9 has potential predictive value for colorectal liver metastases [65]. Other researches also shows that MYH9 was involved in cancer cells death resistance and promotes metastasis [66, 67]. eEF2 and phosphorylated eEF2 are prognostic indicators of HCC patient survival [68], and that eEF2 kinase promotes HCC angiogenesis and tumor progression through SP1/KLF5-mediated VEGF expression [69]. Modulation of eEF2 and its kinases is therefore a potential drug target for cancer therapy. Alkaloids in Coptidis rhizome suppresses eEF2 activity, and then inhibits tumor growth and angiogenesis in animal experiments, suggesting anti-hepatoma efficacy [70]. In addition, eEF2 was also related to chemoresistance in malignant tumors [71, 72]. Tax1 binding protein 3, also known as TIP-1, is widely involved in biological processes through selective protein interactions. TAX1BP3 expression levels are increased in human invasive breast cancer, and contribute to cell adhesion to extracellular matrix, invasion and lung metastasis [73]. TAX1BP3 expression could facilitate angiogenesis and tumor formation of human glioblastoma cells and were closely correlated with the prognosis of glioblastoma patients [74], suggesting that TAX1BP3 can be regarded as a prognostic marker for human glioblastoma. Additionally, TAX1BP3 can be a therapy target to regulate chemosensitivity of gastric cancer cells to 5-FU [75]. Accordingly, it is reasonable to assume that these membrane proteins might be involved in matrix stiffness-mediated metformin resistance in HCC. However, few studies related to the six membrane proteins above were focused on the biomechanics impact within tumor microenvironment on therapeutic resistance. Thus, little is known about whether changes and remodeling of matrix stiffness-induced membrane proteins modulates the malignant characteristics of HCC cells. The signaling pathways which the above membrane proteins might be mediated or participated predicted by GO enrichment analysis were also seldomly reported in cancer chemoresistance previously, which deserves further study. To search for the membrane proteins related to matrix stiffness-induced therapeutic resistance and explore the potential mechanisms, which is the purpose of our study.

## Conclusions

In summary, we identified some differential membrane proteins and membrane associated proteins related to matrix stiffness-mediated metformin resistance in HCC cells in this study. Six candidate membrane proteins can reflect the response of HCC cells under high stiffness stimulation to metformin intervention, which deserve to be investigated in the future.

### Supplementary Information

**Additional file 1: Table S1. **Ingredients of polyacrylamide gel substrates with variable stiffness.

**Additional file 2: Figure S1. **Efficiency and quality identification of membrane protein extraction. (A) The approximate location and range of membrane protein molecules performed by polyacrylamide gel electrophoresis with Coomassie brilliant blue staining. (B)(i, ii) Western blot were performed on the six groups of membrane proteins and cytoplasmic proteins.

**Additional file 3: Table S2. **A list of all identified proteins.

**Additional file 4: Table S3. **Differential membrane proteins and membrane associated proteins.

**Additional file 5: Figure S2. **Six typical expression patterns reflect the increase degree of membrane proteins. iTRAQ tags 113, 114, 115, 116, 117 and 118 represent NC-L, NC-M, NC-H, Met-L, Met-M and Met-H, respectively.

**Additional file 6: Figure S3. **Six typical expression patterns reflect the decrease degree of membrane proteins. iTRAQ tags 113, 114, 115, 116, 117 and 118 represent NC-L, NC-M, NC-H, Met-L, Met-M and Met-H, respectively.

## Data Availability

The datasets used and/or analysed during the current study are available from the corresponding author on reasonable request.

## Abbreviations

HCC: Hepatocellular carcinoma
iTRAQ: Isobaric tags for relative and absolute quantification
LC–MS/MS: Liquid chromatography-tandem mass spectrometry
ANXA2: Annexin A2
FLNA: Filamin-A
MSN: Moesin
MYH9: Myosin-9
eEF2: Eukaryotic Elongation Factor 2
TAX1BP3: Tax1-binding protein 3
EMT: Epithelial-mesenchymal transition
FBS: Fetal bovine serum
SDS-PAGE: Sodium dodecyl sulphate–polyacrylamide gel electrophoresis
PVDF: Polyvinylidene difluoride
PPI: Protein–protein interactions
IPA: Ingenuity Pathway Analysis
GO: Gene Ontology
