# Supplementary material for: Analysis of differential membrane proteins related to matrix stiffness-mediated metformin resistance in hepatocellular carcinoma cells
Source: Proteome Sci. 2023 Sep 22;21:14. doi: 10.1186/s12953-023-00216-7 (PMC10517517; doi:10.1186/s12953-023-00216-7)
Supplement: Supplementary file 1 — Additional file 1: Table S1. Ingredients of polyacrylamide gel substrates with variable stiffness. [file 12953_2023_216_MOESM1_ESM.docx]

**Table S1 Ingredients of polyacrylamide gel substrates with variable stiffness.**

|  | 30%Acr（μl） | 2%Bis（μl） | 10%APS（μl） | TEMED（μl） | Serum-free medium（μl） | Total Volume (μl) |
| --- | --- | --- | --- | --- | --- | --- |
| L（6 kPa） | 2000 | 270 | 60 | 6 | 3664 | 6000 |
| M（10 kPa） | 2000 | 570 | 60 | 6 | 3364 | 6000 |
| H（16 kPa） | 2000 | 1500 | 60 | 6 | 2434 | 6000 |
