# Supplementary figures and images for: Analysis of differential membrane proteins related to matrix stiffness-mediated metformin resistance in hepatocellular carcinoma cells
Source: Proteome Sci. 2023 Sep 22;21:14. doi: 10.1186/s12953-023-00216-7 (PMC10517517; doi:10.1186/s12953-023-00216-7)

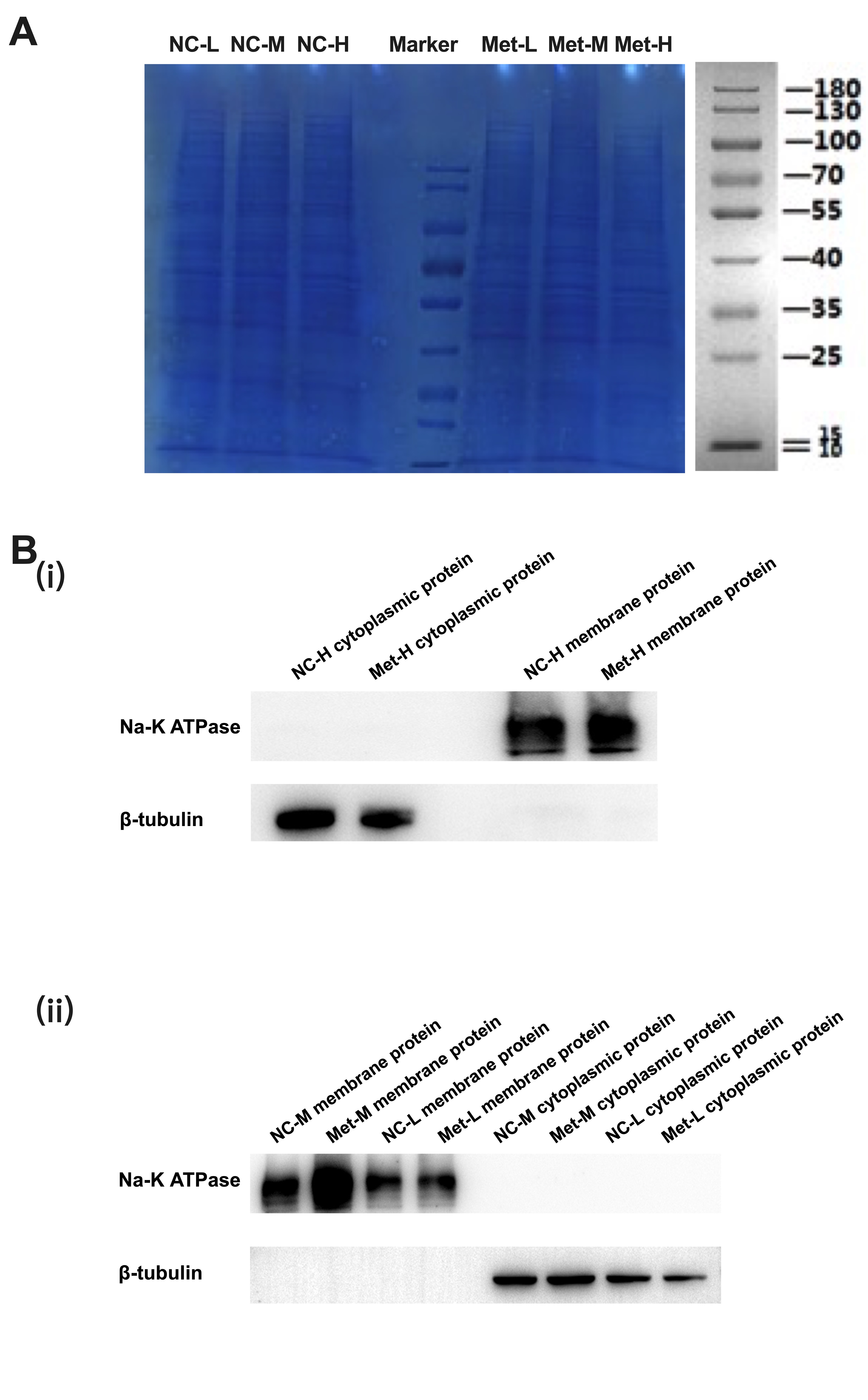

Supplement: Supplementary file 2 — Additional file 2: Figure S1. Efficiency and quality identification of membrane protein extraction. (A) The approximate location and range of membrane protein molecules performed by polyacrylamide gel electrophoresis with Coomassie brilliant blue staining. (B)(i, ii) Western blot were performed on the six groups of membrane proteins and cytoplasmic proteins. [file 12953_2023_216_MOESM2_ESM.jpg]

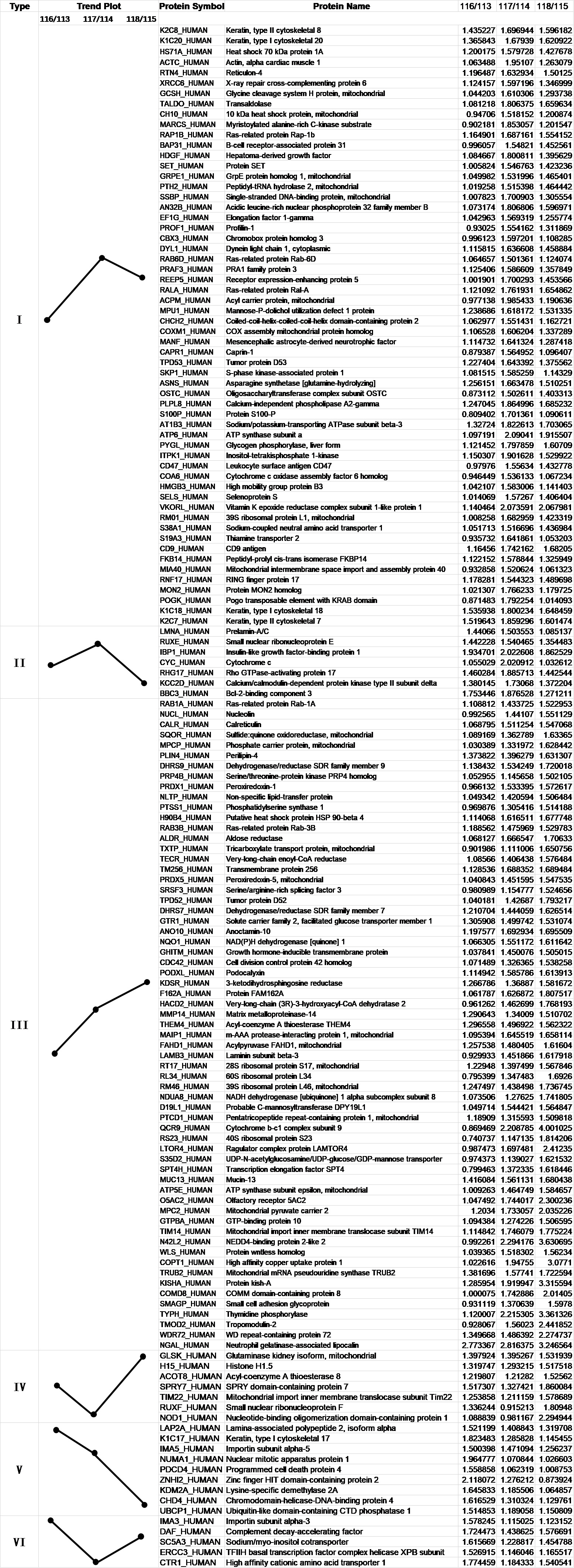

Supplement: Supplementary file 5 — Additional file 5: Figure S2. Six typical expression patterns reflect the increase degree of membrane proteins. iTRAQ tags 113, 114, 115, 116, 117 and 118 represent NC-L, NC-M, NC-H, Met-L, Met-M and Met-H, respectively. [file 12953_2023_216_MOESM5_ESM.jpg]

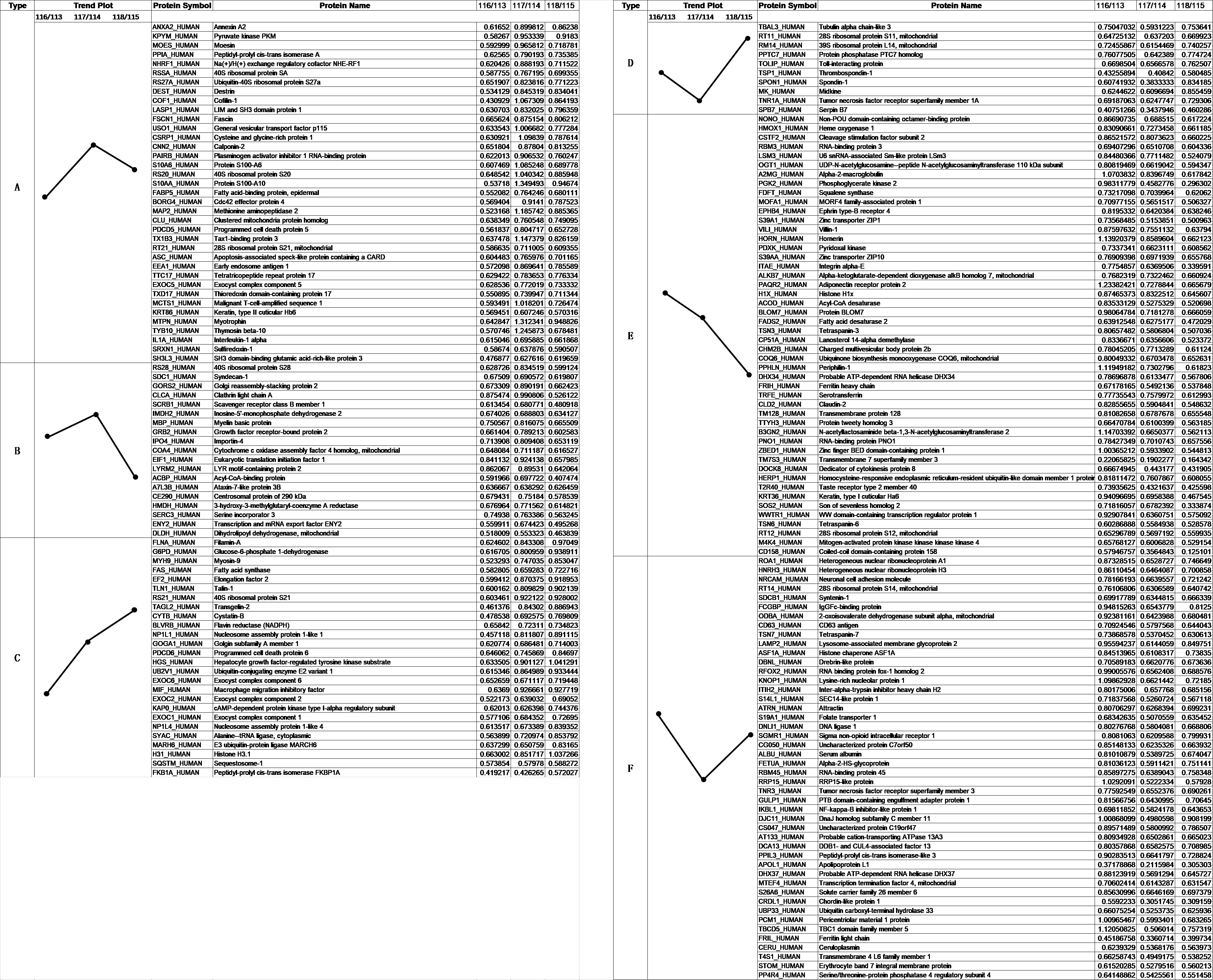

Supplement: Supplementary file 6 — Additional file 6: Figure S3. Six typical expression patterns reflect the decrease degree of membrane proteins. iTRAQ tags 113, 114, 115, 116, 117 and 118 represent NC-L, NC-M, NC-H, Met-L, Met-M and Met-H, respectively. [file 12953_2023_216_MOESM6_ESM.jpg]
